# Impact of Selected Small-Molecule Kinase Inhibitors on Lipid Membranes

**DOI:** 10.3390/ph14080746

**Published:** 2021-07-29

**Authors:** Meike Luck, Markus Fischer, Maximilian Werle, Holger A. Scheidt, Peter Müller

**Affiliations:** 1 Department of Biology, Humboldt-Universität zu Berlin, Invalidenstr. 42, 10115 Berlin, Germany; luckmeik@cms.hu-berlin.de (M.L.); link06@live.de (M.W.); 2 Institute for Medical Physics and Biophysics, Leipzig University, Härtelstr. 16-18, 04107 Leipzig, Germany; markus.fischer@medizin.uni-leipzig.de

**Keywords:** small-molecule kinase inhibitors, lipid membranes, membrane structure, sunitinib, erlotinib, idelalisib, lenvatinib, NMR, fluorescence

## Abstract

Small-molecule protein kinase inhibitors are used for the treatment of various diseases. Although their effect(s) on the respective kinase are generally quite well understood, surprisingly, their interaction with membranes is only barely investigated; even though these drugs necessarily come into contact with the plasma and intracellular membranes. Using biophysical methods such as NMR, ESR, and fluorescence spectroscopy in combination with lipid vesicles, we studied the membrane interaction of the kinase inhibitors sunitinib, erlotinib, idelalisib, and lenvatinib; these drugs are characterized by medium log *p* values, a parameter reflecting the overall hydrophobicity of the molecules, which is one important parameter to predict the interaction with lipid membranes. While all four molecules tend to embed in a similar region of the lipid membrane, their presence has different impacts on membrane structure and dynamics. Most notably, sunitinib, exhibiting the lowest log *p* value of the four inhibitors, effectively influences membrane integrity, while the others do not. This shows that the estimation of the effect of drug molecules on lipid membranes can be rather complex. In this context, experimental studies on lipid membranes are necessary to (i) identify drugs that may disturb membranes and (ii) characterize drug–membrane interactions on a molecular level. Such knowledge is important for understanding the efficacy and potential side effects of respective drugs.

## 1. Introduction

In order to understand the impact of therapeutically applied drugs on cells, the knowledge about interactions of the molecules with cellular membranes is of high relevance. Firstly, plasma membranes can be defined as the primary contact site of the drug with the cell. Secondly, upon drug uptake, interactions with intracellular organelle membranes will occur. It can be presumed that this aspect of cellular drug effect(s) is important for understanding the efficacy and side effects of the respective substances. However, despite this significance, drug–membrane interactions have not been investigated in detail for many existing medications.

One obvious approach to anticipate the membrane interaction of membrane active molecules is to consider their log *p* value, which quantitatively reflects the partition of the respective compound between octanol and water. The higher the log *p* value of a molecule, and thus the affinity to the hydrophobic octanol phase, the higher should also be its tendency to incorporate into membranes and therefore the prospect to cause a membrane impact. The contrary is expected for molecules with low log *p* values. Of course, focusing on log *p* values is a very simplified method and more (physico-chemical) parameters have to be considered to understand drug–membrane interactions on a molecular level. This is in line with the rule of five proposed by Lipinski et al. [1], which includes additional parameters such as hydrogen bond donors and acceptors as well as the molecular mass in order to estimate solubility and permeability in drug discovery and drug development procedures. However, an incorporation of molecules into a certain part of a membrane is not necessarily accompanied by an influence on its properties, such as membrane integrity and structure/dynamics of membrane components. The nature, the number and the localization of drug molecules in the lipid bilayer define the degree of impact and disturbances.

In case of the family of small-molecule kinase inhibitors, the knowledge about membrane impact is still insufficient. That is why we recently performed studies characterizing the interaction of those molecules with lipid membranes. Small-molecule kinase inhibitors are a class of heterogeneous molecules of small size that inhibit the phosphorylation of proteins or lipids by blocking the respective kinases. Since an overregulation of kinase activity may cause various kinds of cancer disease, the usage of small-molecule kinase inhibitors is an established approach in cancer therapy. Currently, 65 FDA-approved small-molecule kinase inhibitors are therapeutically applied (http://www.brimr.org/PKI/PKIs.htm, accessed on 7 March 2021). Comparing the log *p* values, these molecules display astonishing differences covering more than 6 orders of magnitude in the partition equilibrium. In order to investigate the interaction of small-molecule kinase inhibitors with membranes, we have focused recently on molecules with high and low log *p* values, hypothesizing a large membrane impact for the former and a lower one for the latter. Indeed, we found for lapatinib (log *p* = 6.45, calculated with Molinspiration, http://www.molinspiration.com/, accessed on 15 June 2021) compared to tofacitinib (log *p* = 0.45) a deeper embedding into phospholipid membranes and an increased membrane permeability for polar molecules [2]. Consistently, in a comparable study, no disturbance of membrane integrity was observed for the inhibitor ruxolitinib (which has recently been considered for usage in the treatment of COVID-19 [3]) having a moderate log *p* value of 1.83 [4].

**Figure 1:**
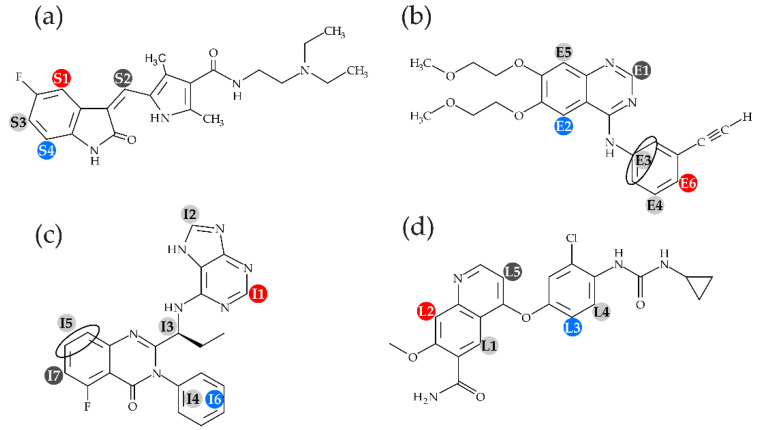
Structures and proton assignment of the researched kinase inhibitors (**a**) suntinib, (**b**) erlotinib, (**c**) idelalisib, (**d**) lenvatinib, as well as the protons used in ^1^H-^1^H MAS NOESY NMR.

In the present work, we studied small-molecule kinase inhibitors with mean log *p* values, i.e., we used the drugs sunitinib (log *p* = 1.95), erlotinib (log *p* = 2.90), idelalisib (log *p* = 3.68), and lenvatinib (log *p* = 4.36) (structures see Figure 1). We note that this ‘mean’ range of log *p* values still represents a larger than 100-fold difference in the partition of respective molecules between octanol and water.

Erlotinib inhibits the tyrosine kinase of the epidermal growth factor receptor (EGFR) and has been used for a couple of years in the treatment of non-small cell lung cancer (NSCLC) [5]. It showed even more promising results than chemotherapy against advanced EGFR mutation-positive non-small-cell lung cancer [6] and is also applied in combination with sunitinib against NSCLC [7]. Sunitinib is a multitarget tyrosine kinase inhibitor that is often used in the treatment of advanced gastrointestinal stromal tumor when resistant to imatinib [8] and also results in lengthened progression-free survival in patients with pancreatic neuroendocrine tumors [9]. Lenvatinib is another tyrosine kinase inhibitor that is effective against thyroid cancer along with broad antitumor and antiangiogenic activity [10]. Idelalisib is a representative of the few drugs inhibiting lipid kinases. It affects phosphatidylinositol-3-kinase activity [11] and is used in the treatment of relapsed chronic lymphocytic leukemia (CLL) in conjunction with rituximab [12] and is recommended for treatment of non-Hodgkin lymphoma [13].

The membrane impact of these kinase inhibitors was investigated using various methods of NMR, ESR, and fluorescence spectroscopy and lipid vesicles containing 1-palmitoyl-2-oleoyl-*sn*-glycero-3-phosphocholine (POPC) and POPC/cholesterol. These two lipids belong to the major lipid species present in biological membranes. The experiments should answer the following questions: (i) Do the molecules incorporate into lipid bilayers? (ii) In which membrane region(s) do the molecules embed? (iii) What influence do the molecules have on membrane structure, dynamics, and integrity?

## 2. Results

### 2.1. Localization of Kinase Inhibitors in the Membrane

^1^H MAS NMR spectroscopy was used to investigate the localization and orientation of the kinase inhibitors in the bilayer of POPC membranes. ^1^H MAS NMR spectra of POPC membranes in the presence of 20 mol% of the molecules are shown in Figure S1. The ^1^H NMR peak assignment for the inhibitors was performed with the help of the SPINUS NMR prediction (http://neural.dq.fct.unl.pt/spinus/, aceeseed on 15 June 2021), as well as ^1^H NMR spectra provided by manufacturers.

Each inhibitor produced unique ^1^H NMR signals besides those of POPC, which allowed to measure cross-relaxation rates of the respective protons to molecular segments of the POPC membrane in ^1^H-^1^H MAS NOESY experiments. The plot of these cross-relaxation rates over the molecular groups of POPC along the membrane normal can be interpreted as a distribution function of the inhibitor protons across the normal membrane. Previous works performed similar approaches for many other small molecules including kinase inhibitors [2,4,14,15]. From these data, conclusions about the membrane position and orientation of the respective molecule can be drawn.

**Figure 2:**
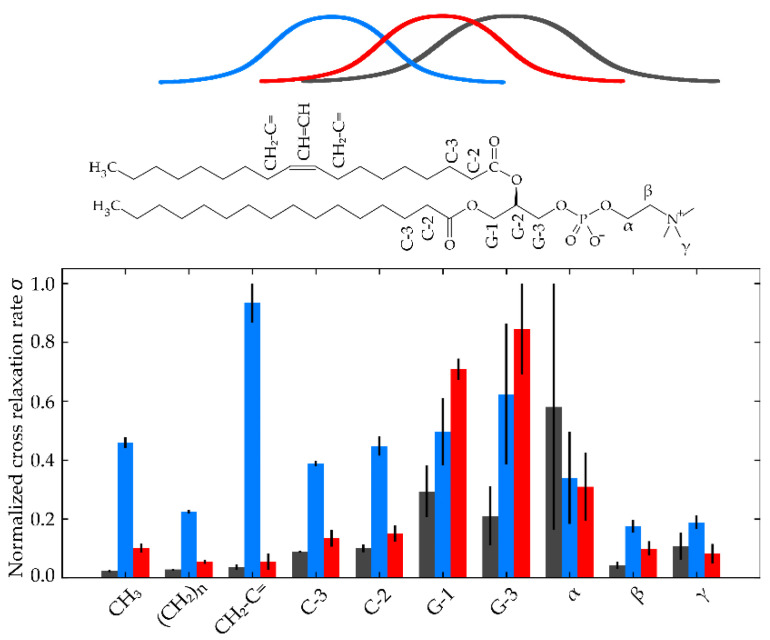
Normalized cross relaxation rates of the protons (I1-red, I6-blue, I7-green, see also Figure 1) of idelalisib with the proton groups of the POPC membrane. The data show that the aromatic ring of the molecule points into the hydrocarbon membrane, while both the naphtalene and the indane-like substructures are oriented towards the aqueous phase. On average, idelalisib is localized in the glycerol region of the membrane, however, it remains mobile throughout the bilayer.

In Figure 2, exemplary for all measured drugs, the obtained cross relaxations rates of three protons of idelalisib (I1, I6 and I7, see Figure 1) over the molecular segments of POPC along the membrane normal are shown. The protons were chosen due to their distribution over the idelalisib molecule and their well resolved cross peaks in the NOESY spectra (see Figure S2). All protons exhibit a relatively broad distribution function, which is a result of the high molecular disorder and the dynamics in lipid membranes. For the protons of I1, the maximum of this distribution is located in the glycerol region of the membrane. While for the protons of the aromatic ring (I6), a somewhat deeper position is observed; for the protons of I7, the maximum is shifted in the direction of the headgroup region. From these data, one can conclude an average location and orientation of idelalisib with the aromatic ring pointing into the hydrocarbon region of the membrane, while the naphtalene and indane-like substructures are oriented towards the aqueous phase.

A similar picture was obtained for the other three inhibitors investigated. All show a mean position in the glycerol region of the phospholipid bilayer (see Figures S3–S5), but having broad distributions along the membrane normal, which indicates high molecular dynamics with regard to rotational and translational mobility. Therefore, it is difficult to determine a clear orientation of these molecules in the lipid membrane.

### 2.2. Influence of Kinase Inhibitors on Headgroup and Acyl Chain Behavior of the Phospholipid Membrane

To investigate the influence of the inhibitor molecules on the structure and dynamics of the surrounding lipids, ^31^P and ^2^H NMR spectroscopy were employed for analyzing the behavior of the phosphatidyl headgroup and the acyl chains of POPC.

Analysis of line shapes of static ^31^P NMR powder spectra (see Figure S6) provides information about the phase state of the phospholipid membrane as well as vesicle orientation and headgroup dynamics. The ^31^P NMR line shapes indicate that the lamellar bilayer phase of the POPC vesicles is maintained in presence of each of the inhibitors. Except for sunitinib, all other drugs have little to no effect on the mobility of the headgroup as indicated by very similar values of the chemical shift anisotropy (CSA). Sunitinib slightly raises the CSA, which indicates a more constrained phosphatidyl headgroup or changes in head group orientation, probably due to a localization of sunitinib near the aqueous phase. 

**Figure 3:**
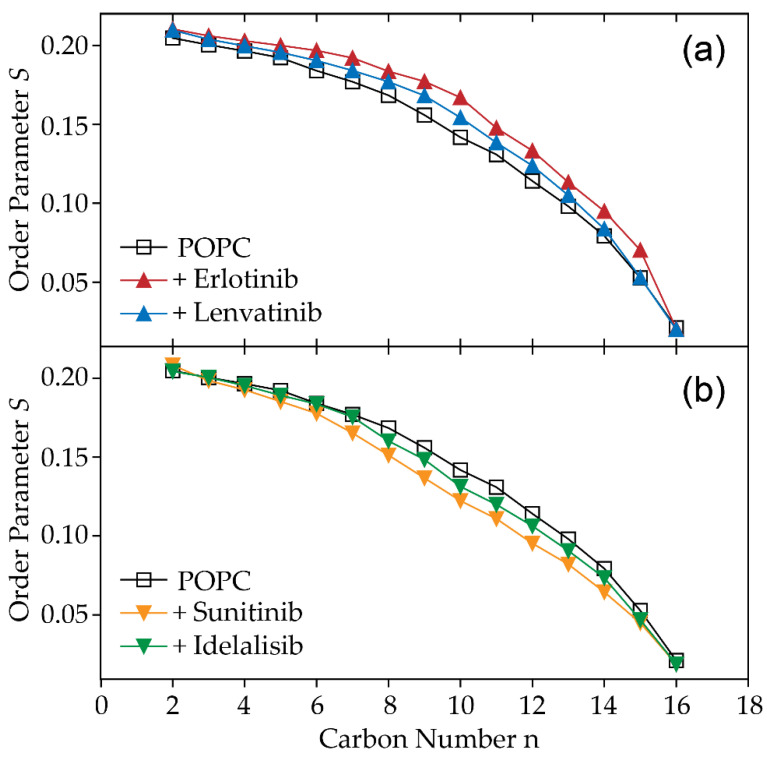
^2^H NMR smoothed order parameter profiles of the *sn*-1 chain perdeuterated analogue of POPC (POPC-*d*_31_) both in the absence and in the presence of 20 mol% of each of the inhibitors. While lenvatinib and erlotinib (**a**) raise the order parameter, indicating stiffening of the membrane, idelalisib and sunitinib (**b**) lower the order parameter along the chain.

Static ^2^H NMR spectroscopy was used to compare the impact of the different inhibitors on acyl chain behavior, as well as on lipid chain packing. The ^2^H NMR Pake spectra of the lamellar liquid crystalline lipid membrane were measured in the presence of each inhibitor and are shown in Figure S7. Smoothed chain order parameters calculated from the depaked spectra are shown in Figure 3. The chain extent L_c_* was calculated according to Petrache et al. [16].

Both erlotinib and lenvatinib raise the acyl chain order parameter (see Figure 3a), which indicates an increase in lipid chain packing, also reflected by the increase in chain length L_c_* (see Table 1). The other two inhibitors sunitinib and idelalisib decrease the ordering of the lipid chains, with a more pronounced effect in the presence of sunitinib (see Figure 3b). This corresponds to shorter chain lengths L_c*_ and a decrease in the lipid change packing. The four inhibitors can therefore be divided in two subgroups, either increasing or decreasing the lipid membrane density.

### 2.3. Influence of Kinase Inhibitors on Transmembrane Permeation of Anions

**Figure 4:**
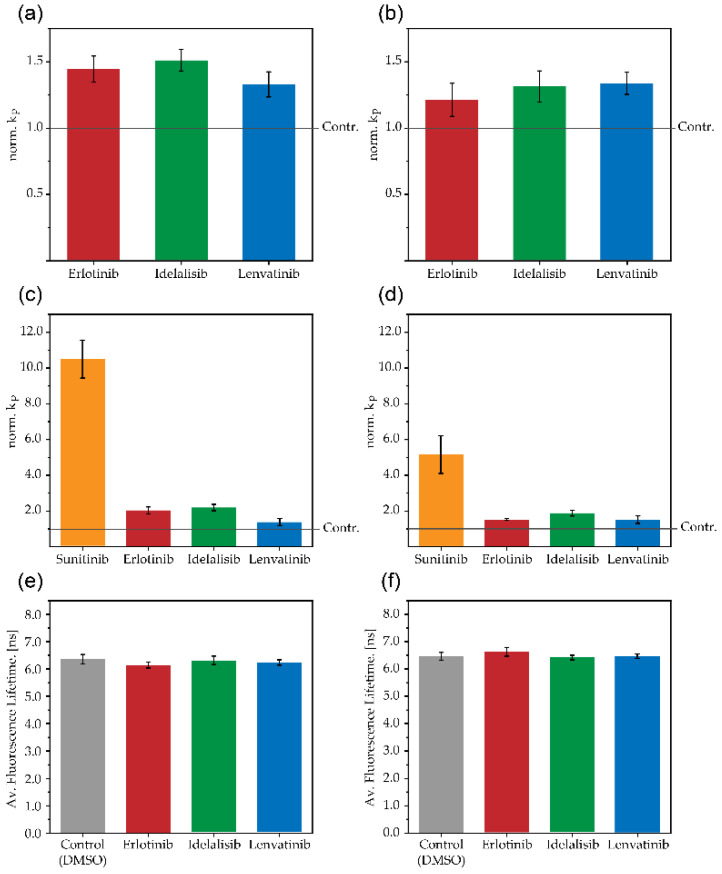
Influence of small-molecule kinase inhibitors on membrane properties. The membrane permeation of dithionite for POPC LUVs (**a**) and POPC/cholesterol (4:1) LUVs (**b**), and of ascorbate for POPC LUVs (**c**) and POPC/cholesterol (4:1) LUVs (**d**) was measured in the absence and presence of drugs by fluorescence and ESR spectroscopy. From experimental reduction kinetics of NBD-PC by dithionite or of spin-labeled-lipids by ascorbate measured at 37 °C, the rate constants (k_p_) for either anion were determined. The k_p_ values in in the presence of respective drugs (L:D = 5:1) were normalized to those determined in the absence of the drugs (only addition of DMSO). The mean +/− SE of three independent experiments are shown. Moreover, the average fluorescence lifetime (τ_av_) of NBD-PC labelled POPC (**e**) and POPC/cholesterol (4:1) (**f**) LUVs was measured in the absence (only addition of DMSO) or in the presence of the given drugs (L:D = 2.5:1) at 25 °C. The data represent the mean +/− SD of at least three independent experiments.

The impact of kinase inhibitors on membrane structure was determined by measuring their influence on membrane permeation of the ions dithionite and ascorbate using a fluorescence- and ESR-based assay, respectively (see Section 4. This permeation is normally rather slow. A disturbance of membrane integrity e.g., due to the presence of membrane-inserting molecules, is reflected by an accelerated ion permeation. The experiments were performed for POPC and POPC/cholesterol (4:1) large unilamellar vesicles (LUVs), which were labeled with 1-palmitoyl-2-(12-[N-(7-nitrobenz-2-oxa-1,3-diazol-4-yl)amino]dodecanoyl)-*sn*-glycero-3-phosphocholine (NBD-PC) or 1-palmitoyl-2-(4-doxylpentanoyl)-*sn*-glycero-3-phosphocholine (SL-PC). The fluorescence data show that lenvatinib, erlotinib, and idelalisib caused a low or no increase of the dithionite permeation constant for both vesicle types at a lipid to drug ratio (L:D = 5:1) (see Figure 4a,b). Similar data were obtained in the presence of larger drug concentrations (L:D = 2.5:1), with the exception of lenvatinib in POPC LUVs, which showed about threefold accelerated NBD-reduction kinetics (see Figure S8). Unfortunately, this assay could not be performed for sunitinib. This molecule has strong light absorption characteristics in the same wavelength range as the NBD moiety [17], impeding a successful recording of NBD fluorescence in the presence of sunitinib. However, using the ESR assay, which does not depend on light absorption, we found in the presence of sunitinib a significant increase of the ascorbate permeation rate (see Figure 4c,d). This impact was twofold larger in POPC vesicles compared to cholesterol-containing LUVs. In agreement with the dithionite assay, no or only a moderate increase of the ascorbate permeation rate constant was observed in the presence of lenvatinib, erlotinib or idelalisib.

### 2.4. Influence of Kinase Inhibitors on Fluorescence Lifetime of NBD Labelled Vesicles

The measurement and analysis of the fluorescence lifetime of fluorescent lipids may provide information about the influence of membrane embedded molecules on their molecular environment [18]. The fluorescence life time of NBD-PC was measured in POPC and in POPC/cholesterol (4:1) LUVs without and with the addition of the respective kinase inhibitors. From the kinetics, an average lifetime (τ_av_) was determined (see Section 4. No changes of τ_av_ were detected in the presence of erlotinib, idelalisib, or lenvatinib in comparison to control vesicles even at a rather large drug concentration (L:D = 2.5:1) (see Figure 4e,f). Again, these experiments could not be performed for sunitinib. 

## 3. Discussion

In the present study, we investigated the interaction of the small-molecule kinase inhibitors sunitinib, erlotinib, idelalisib, and lenvatinib with membranes using various biophysical methods. Compared to other small-molecule kinase inhibitors, these therapeutically applied drug molecules have mean log *p* values in common, a parameter describing the overall molecular hydrophobicity. It has been shown that small-molecule kinase inhibitors with large and small log *p* values are capable of binding and interacting to/with lipid membranes ([2], Heerklotz and Scheidt, unpublished data). Therefore, such interactions have to be assumed also for the inhibitors investigated in this study.

Using ^1^H MAS NMR spectroscopy, we found that the four investigated molecules embed mainly into the glycerol region of the phospholipid bilayer with similar distributions of their molecule segments along the phospholipid structure, though with a broad variance along the membrane normal. Some molecular segments also show stronger interactions with the C-2 and C-3 (sunitinib, erlotinib and lenvatinib) or the CH=CH segments of POPC (idelalisib), reflecting a somewhat deeper penetration into the upper hydrophobic core of the lipid membrane. Nevertheless, due to the inherent molecular disorder and internal mobility of lipid membranes, all molecules possess large degrees of rotational and translational freedom within the membrane, indicating a lack of clear and fixed orientation within the bilayer.

Notably, despite such a similar membrane embedding, the kinase inhibitors investigated have different impacts on membrane structure and dynamics. While two drugs, i.e., erlotinib and lenvatinib, increase the lipid chain packing, the presence of sunitinib and idelalisib causes a decrease of this parameter. The latter lowering effect is mainly observed in the middle and lower segments of the fatty acyl chain, which seems to contradict the preferential incorporation of the molecules into the upper bilayer region. An explanation might be that the incorporation of drugs into this membrane section induces a certain “void” space below, allowing greater freedom of movement to the acyl chains. In addition, the high motional dynamics of the molecules observed along the normal membrane could also cause an effect in deeper membrane regions.

The kinase inhibitors have different degrees of influence on membrane integrity as concluded from their impact on ion permeation. While a treatment with sunitinib results in a significant disturbance of the membrane as seen from the increased permeation rate constants of ascorbate in POPC as well as in POPC/cholesterol membranes, the other molecules either not at all or only slightly affect ascorbate and dithionite permeability. Unfortunately, sunitinib could not be investigated with the alternative dithionite assay (see above) to confirm the strong impact of this inhibitor on membrane integrity as observed in the ESR-based assay. Interestingly, the presence of sunitinib also results in the comparatively largest decrease of lipid chain order parameters compared to the other molecules.

Furthermore, erlotinib, idelalisib, and lenvatinib have no influence on the fluorescence lifetime of NBD-PC, indicating no or low disturbances of the membrane structure in the region of the NBD moiety. This is in line with the other data of this study, proposing a similar membrane embedding and no or low effects on lipid chain order in the upper bilayer region for these molecules. Note that the NBD-PC used here bears the label moiety at the end of a long (C12) fatty acyl chain, which might suggest a localization of the NBD group in the deeper hydrophobic membrane region. However, it has been shown that the NBD group, due to its polarity, loops back to the water/lipid interface, thereby sensing the conditions of this membrane region [19,20,21]. Since these experiments also could not be performed for sunitinib, a potential effect of this molecule on the surrounding of the NBD-group could not be verified.

The different extents of impact of the four kinase inhibitors on membrane structure and integrity found in this study underline the necessity to consider various molecular parameters for explaining drug–membrane interactions [1,22]. Describing the present data solely on the basis of log *p* values, the lowest impact on membranes would be expected for sunitinib. However, this drug, while displaying a similar embedding into the lipid bilayer like the other inhibitors, causes the strongest influence on membrane permeation. Notably, we recently found that the kinase inhibitor ruxolitinib, which has a log *p* value comparable to sunitinib, does not influence lipid membranes similarly at identical conditions [4]. Molecular parameters that additionally have to be considered to understand the physiological impact of drugs, are, among others, the molecular weight, steric orientations, the dipolar moment, the number and spatial arrangement of hydrogen bond donor and acceptor atoms, and the topological polar surface area [1]. E.g., it is conceivable that the presence of hydrogen bond donors and acceptors allows the formation of hydrogen bonds between a drug molecule and surrounding lipids in the membrane, which may also influence and define the character of drug embedding into the bilayer and the interaction with the surrounding lipidic phase [23,24].

Therefore, experimentally investigating the interaction of (medically applied) drugs with lipid membranes supports our knowledge about physiological drug-mediated effects [25]. Although those studies are not able to present alternative mechanism(s) of drug efficacy, i.e., of their specific impact on the respective kinase, they might help to understand and to overcome unspecific side effects of the drugs, which are as follows:

Firstly, the application of drugs is often accompanied by (unspecific) cytotoxic effects due to invasive impacts on enzymes, organelles and/or membranes, which have also been shown for the molecules investigated in this study [26,27,28,29,30]. Such toxicities might be (partially) caused by a drug-mediated cell lysis due to a perturbation of the plasma membrane, which could be reflected by an increased ion permeability across (lipid) membranes as found here for sunitinib. Secondly, a drug-mediated influence on membrane properties, like membrane fluidity or lateral lipid arrangement, may modify the activity of membrane-bound enzymes [31,32,33], which might have harmful consequences on the cellular level. Thirdly, it was shown that tyrosine kinase inhibitors are substrates for and/or inhibitors of various ATP-binding cassette (ABC) transporters, which require an interaction between both molecules within the membrane. The knowledge of this process is important for understanding the pharmacokinetics of drug accumulation within cells (see below) [34,35,36].

Moreover, drug–membrane studies contribute to understanding and improving the cellular uptake of the respective substances. For many molecules, the precise uptake mechanisms, i.e., whether they enter cells by passive diffusion or by active transport processes like endocytosis or membrane transporters, are unknown. Although some studies have dealt with this topic, the cellular import process(es) is/are not yet identified for the inhibitors used here to the best of our knowledge [37,38,39,40,41]. An effective membrane binding of a drug may support its passive transmembrane diffusion, arguing for an uptake via diffusion. To the contrary, a low water solubility of hydrophobic molecules may hinder their effective entrance into target cells. To improve a cellular uptake, e.g., in therapeutic application, those drugs are incorporated into supra-molecular structures to facilitate their uptake via endocytosis [42,43,44,45,46,47,48,49].

To summarize, to understand unspecific cellular effects of drugs on a molecular level, the impact of the molecules on membranes should be considered. For that, experimental studies on lipid membranes may help to identify drugs with an ability to effectively disturb membranes. With rising sets of data and of knowledge about potential structural changes in the lipid bilayer, the results might be correlated to side effects and prospectively support the drug development processes.

## 4. Materials and Methods

### 4.1. Chemicals

The lipids POPC, cholesterol, NBD-PC, and POPC-*d*_31_ were purchased from Avanti Polar Lipids, Inc. (Alabaster, AL, USA). SL-PC was prepared as described previously [50]. Kinase inhibitors were acquired from Hycultec GmbH (Beutelsbach, Germany), while all other chemicals were attained from Sigma-Aldrich (Taufkirchen, Germany) and used without further purification. For the experiments, stock solutions of the drugs were prepared in DMSO and stored at −80 °C.

### 4.2. Preparation of LUVs

Preparation of LUVs was performed by the extrusion method as described by [51]. Briefly, aliquots of the respective lipids were dissolved in chloroform and dried in a rotating round-bottom flask under vacuum until a lipid film was formed. Subsequently, lipids were resuspended in a small volume of ethanol (final ethanol concentration was below 1% (*v*/*v*) and HEPES buffered saline (HBS, 150 mM NaCl and 10 mM HEPES, pH 7.4) was added to reach a final lipid concentration of 1 mM. After vortexing, the suspension was subjected to six freeze-thaw cycles. Finally, LUVs were generated by extrusion of the lipid suspension 10 times through 0.1 μm polycarbonate filters at 50 °C (extruder from Lipex Biomembranes Inc., Vancouver, BC, Canada; filters from Costar, Nucleopore, Tübingen, Germany).

### 4.3. Sample Preparation for NMR Experiments

Both lipids and inhibitors were dissolved in chloroform and mixed together in an 8:2 molar ratio, after which the solvent was evaporated. The samples were re-dissolved in cyclohexane and lyophilized overnight at high vacuum. A fluffy white powder was obtained and then hydrated with 50 wt% H_2_O buffer solution (100 mM NaCl, 10 mM HEPES, pH 7.4) for ^2^H and ^31^P NMR experiments, or D_2_O buffer for ^1^H MAS NMR experiments. The samples were equilibrated by multiple freeze-thaw cycles and gentle centrifugation, and then transferred to 4 mm HR MAS rotors with spherical Kel-F inserts.

### 4.4. H NMR and ^31^P NMR Spectroscopy

Static solid-state NMR spectra were acquired on both a Bruker DRX 300 (for ^2^H), as well as a 750 MHz Bruker Avance I (for ^31^P) NMR spectrometer (Bruker Biospin GmbH, Rheinstetten, Germany). All measurements were performed at 303 K.

A Hahn echo sequence was used for the ^31^P NMR experiments, with a relaxation delay of 2.5 s, ^1^H decoupling, and a 90° pulse of about 3.5 µs. Approximately 20 k scans were conducted, and a line broadening of 100 Hz was applied. Simulations of the line shapes were done in a Python 3.7 script.

The ^2^H NMR spectra were acquired with a phase-cycled quadrupolar echo sequence [52] with a recycle delay of 1 s. The two π/2 pulses of about 4 µs were staggered by a 50 µs delay and, depending on the sample, 8 k to 12 k scans were conducted. After depaking [53] the spectra, smoothed order parameter profiles were calculated according to [54].

### 4.5. H MAS NMR Spectroscopy

^1^H MAS NMR measurements were conducted on a Bruker Avance III 600 MHz spectrometer equipped with a 4 mm HR-MAS probe operating at a MAS frequency of 6 kHz with a π/2 pulse length of 4 µs. 

For all ^1^H NMR spectra, the chemical shift of the terminal methyl group of the POPC was calibrated to 0.885 ppm relative to TMS. Five different two-dimensional NOESY spectra were acquired with mixing times between 0.1 ms and 500 ms, a relaxation delay of 3.3 s, and 256 datapoints in the indirect dimension with at least 32 scans each.

The volume of the resulting cross- and diagonal peaks was integrated with the Bruker Topspin 4.0.6 software and cross-relaxation rates were calculated from fits of NOE build-up curves according to the spin pair model of Scheidt and Huster [14].

### 4.6. Measurement of Membrane Permeation of Polar Molecules

Two assays were applied in order to measure the impact of drugs on membrane structure, determining the influence of kinase inhibitors on the permeation of anions, i.e., dithionite and ascorbate, across membranes. The assays employ the property of dithionite and ascorbate to rapidly reduce and irreversibly quench the fluorescence of the NBD group and the ESR signal intensity of spin-labeled lipids, respectively [15,55,56,57]. When the labeled lipids are incorporated into unilamellar lipid vesicles, the anions quench the signal intensities of the respective labeled lipids in the outer membrane leaflet, which is observed by a rapid initial decrease of signal intensity. Subsequently, the fluorescence or ESR signal intensity decays slowly due to a slow permeation of dithionite/ascorbate across the membrane, reacting with the labeled lipids in the inner membrane leaflet. The rate constant for the slow decrease (k_p_) (i.e., permeation of dithionite/ascorbate across the membrane) was used as parameter for membrane integrity [58,59]. The values of k_p_ in the presence of the drugs were normalized to the respective values in the absence of drugs and in the presence of an equivalent volume of DMSO (solvent of drugs).

### 4.7. Measurement of Fluorescence Lifetime

POPC LUVs containing 1 mM lipid and 0.5 mol% NBD-PC were mixed in a cuvette with the respective kinase inhibitor or DMSO (same volume as used for the drugs). Fluorescence lifetimes on LUVs were measured by time-correlated single photon counting using a FluoTime200 Time-resolved Spectrometer (Picoquant, Berlin, Germany). A 467 nm laser was used for excitation, whereas emission was detected at 540 nm. Data were acquired up to 20,000 counts based on the maximum of the fluorescence lifetime decay curve. The lifetime decay kinetics for both sets of experiments were fitted with two exponential components. From these fittings, an average fluorescence lifetime (τ_av_) was determined according to Equation (1):

(1) $$\tau_{\mathrm{av}}=\frac{\sum_{i}\alpha_{i}\cdot \tau_{i}}{\sum_{i}\alpha_{i}}$$

## Figures and Tables

**Table 1:** The ^31^P NMR chemical shift anisotropy (CSA), the average ^2^H NMR order parameter <S>, and the calculated chain length L_C_* of the POPC *sn-1* acyl chain for pure POPC-*d*_31_ and for POPC-*d*_31_ in the presence of the respective inhibitor.

	^31^P CSA (ppm)	L_c_*[Å]	<S>
POPC	45	11.0	0.144
+Erlotinib	45	11.6	0.154
+Lenvatinib	44	11.2	0.147
+Sunitinib	47	10.6	0.130
+Idelalisib	44	10.8	0.136

## Data Availability

Data are available within this article and in the associated Supplemental Materials.

## Supplementary Materials

The following are available online at https://www.mdpi.com/article/10.3390/ph14080746/s1, Figure S1: ^1^H MAS NMR spectra of POPC in the presence of 20 mol% of each inhibitor, Figure S2: Counter plot of the aromatic region of the ^1^H MAS NOESY spectrum of 20 mol% idelalisib in a POPC membrane, Figure S3: Normalized cross relaxation rates of the protons (E1, E2, and E6) of erlotinib, Figure S4: Normalized cross relaxation rates of the protons (L2, L3, and L5) of lenvatinib, Figure S5: Normalized cross relaxation rates of the protons (S1, S2, and S4) of sunitinib, Figure S6: ^31^P NMR powder spectra of both pure POPC and within and without presence of 20 mol% of each inhibitor, Figure S7: Static ^2^H NMR powder spectra of both pure POPC-*d*_31_, and POPC-*d*_31_ in presence of 20 mol% inhibitor, Figure S8: Influence of small-molecule kinase inhibitors on membrane integrity.

## References

[1] Lipinski C.A., Lombardo F., Dominy B.W., Feeney P.J. (2001). Experimental and computational approaches to estimate solubility and permeability in drug discovery and development settings. Adv. Drug Deliv. Rev..

[2] Haralampiev I., Alonso de Armiño D.J., Luck M., Fischer M., Abel T., Huster D., Di Lella S., Scheidt H.A., Müller P. (2020). Interaction of the small-molecule kinase inhibitors tofacitinib and lapatinib with membranes. Biochim. Biophys. Acta.

[3] Stebbing J., Phelan A., Griffin I., Tucker C., Oechsle O., Smith D., Richardson P. (2020). COVID-19: Combining antiviral and anti-inflammatory treatments. Lancet Infect. Dis..

[4] Fischer M., Luck M., Werle M., Scheidt H.A., Müller P. (2020). Binding of the small-molecule kinase inhibitor ruxolitinib to membranes does not disturb membrane integrity. Biochem. Biophys. Rep..

[5] Bareschino M.A., Schettino C., Troiani T., Martinelli E., Morgillo F., Ciardiello F. (2007). Erlotinib in cancer treatment. Ann. Oncol..

[6] Rosell R., Carcereny E., Gervais R., Vergnenegre A., Massuti B., Felip E., Palmero R., Garcia-Gomez R., Pallares C., Sanchez J.M. (2012). Erlotinib versus standard chemotherapy as first-line treatment for European patients with advanced EGFR mutation-positive non-small-cell lung cancer (EURTAC): A multicentre, open-label, randomised phase 3 trial. Lancet Oncol..

[7] Scagliotti G.V., Krzakowski M., Szczesna A., Strausz J., Makhson A., Reck M., Wierzbicki R.F., Albert I., Thomas M., Miziara J.E.A. (2012). Sunitinib plus erlotinib versus placebo plus erlotinib in patients with previously treated advanced non–small-cell lung cancer: A phase III trial. J. Clin. Oncol..

[8] Demetri G.D., van Oosterom A.T., Garrett C.R., Blackstein M.E., Shah M.H., Verweij J., McArthur G., Judson I.R., Heinrich M.C., Morgan J.A. (2006). Efficacy and safety of sunitinib in patients with advanced gastrointestinal stromal tumour after failure of imatinib: A randomised controlled trial. Lancet.

[9] Raymond E., Dahan L., Raoul J.-L., Bang Y.-J., Borbath I., Lombard-Bohas C., Valle J., Metrakos P., Smith D., Vinik A. (2011). Sunitinib malate for the treatment of pancreatic neuroendocrine tumors. N. Engl. J. Med..

[10] Cabanillas M.E., Habra M.A. (2016). Lenvatinib: Role in thyroid cancer and other solid tumors. Cancer Treat. Rev..

[11] Akinleye A., Avvaru P., Furqan M., Song Y., Liu D. (2013). Phosphatidylinositol 3-kinase (PI3K) inhibitors as cancer therapeutics. J. Hematol. Oncol..

[12] Furman R.R., Sharman J.P., Coutre S.E., Cheson B.D., Pagel J.M., Hillmen P., Barrientos J.C., Zelenetz A.D., Kipps T.J., Flinn I. (2014). Idelalisib and rituximab in relapsed chronic lymphocytic leukemia. N. Engl. J. Med..

[13] Graf S.A., Gopal A.K. (2016). Idelalisib for the treatment of non-Hodgkin lymphoma. Expert Opin. Pharmacother..

[14] Scheidt H.A., Huster D. (2008). The Interaction of small molecules with phospholipid membranes studied by 1H NOESY NMR under Magic-Angle Spinning. Acta Pharmacol. Sin..

[15] Haralampiev I., Scheidt H.A., Abel T., Luckner M., Herrmann A., Huster D., Müller P. (2016). The interaction of sorafenib and regorafenib with membranes is modulated by their lipid composition. Biochim. Biophys. Acta.

[16] Petrache H.I., Dodd S.W., Brown M.F. (2000). Area per lipid and acyl length distributions in fluid phosphatidylcholines determined by (2)H NMR spectroscopy. Biophys. J..

[17] Nowak-Sliwinska P., Weiss A., van Beijnum J.R., Wong T.J., Kilarski W.W., Szewczyk G., Verheul H.M.W., Sarna T., van den Bergh H., Griffioen A.W. (2015). Photoactivation of lysosomally sequestered sunitinib after angiostatic treatment causes vascular occlusion and enhances tumor growth inhibition. Cell Death Dis..

[18] Bastos A.E.P., Scolari S., Stöckl M., de Almeida R.F.M. (2012). Chapter three—Applications of fluorescence lifetime spectroscopy and imaging to lipid domains In Vivo. Meth. Enzymol..

[19] Huster D., Müller P., Arnold K., Herrmann A. (2001). Dynamics of membrane penetration of the fluorescent 7-nitrobenz-2-oxa-1,3-diazol-4-yl (NBD) group attached to an acyl chain of phosphatidylcholine. Biophys. J..

[20] Loura L.M.S., Ramalho J.P.P. (2007). Location and dynamics of acyl chain NBD-labeled phosphatidylcholine (NBD-PC) in DPPC bilayers. A molecular dynamics and time-resolved fluorescence anisotropy study. Biochim. Biophys. Acta.

[21] Mukherjee S., Raghuraman H., Dasgupta S., Chattopadhyay A. (2004). Organization and dynamics of N-(7-nitrobenz-2-oxa-1,3-diazol-4-yl)-labeled lipids: A fluorescence approach. Chem. Phys. Lipids.

[22] Dias M., Hadgraft J., Lane M.E. (2007). Influence of membrane–solvent–solute interactions on solute permeation in model membranes. Int. J. Pharm..

[23] Roskoski R. (2016). Classification of small molecule protein kinase inhibitors based upon the structures of their drug-enzyme complexes. Pharmacol. Res..

[24] Roskoski R. (2021). Properties of FDA-approved small molecule phosphatidylinositol 3-kinase inhibitors prescribed for the treatment of malignancies. Pharmacol. Res..

[25] Alves A.C., Ribeiro D., Nunes C., Reis S. (2016). Biophysics in cancer: The relevance of drug-membrane interaction studies. Biochim. Biophys. Acta.

[26] Porta C., Paglino C., Imarisio I., Bonomi L. (2007). Uncovering Pandora’s vase: The growing problem of new toxicities from novel anticancer agents. The case of sorafenib and sunitinib. Clin. Exp. Med..

[27] McMullen C.J., Chalmers S., Wood R., Cunningham M.R., Currie S. (2020). Sunitinib and imatinib display differential cardiotoxicity in adult rat cardiac fibroblasts that involves a role for calcium/calmodulin dependent protein kinase II. Front. Cardiovasc. Med..

[28] Damaraju V.L., Kuzma M., Cass C.E., Putman C.T., Sawyer M.B. (2018). Multitargeted kinase inhibitors imatinib, sorafenib and sunitinib perturb energy metabolism and cause cytotoxicity to cultured C2C12 skeletal muscle derived myotubes. Biochem. Pharmacol..

[29] Brown J.R. (2019). Phosphatidylinositol 3 kinase δ inhibitors: Present and future. Cancer J..

[30] Paech F., Bouitbir J., Krähenbühl S. (2017). Hepatocellular toxicity associated with tyrosine kinase inhibitors: Mitochondrial damage and inhibition of glycolysis. Front. Pharmacol..

[31] Peters G.H., Bywater R.P. (2001). Influence of a lipid interface on protein dynamics in a fungal lipase. Biophys. J..

[32] Rujiviphat J., Meglei G., Rubinstein J.L., McQuibban G.A. (2009). Phospholipid association is essential for dynamin-related protein Mgm1 to function in mitochondrial membrane fusion. J. Biol. Chem..

[33] Nasr M.L., Shi X., Bowman A.L., Johnson M., Zvonok N., Janero D.R., Vemuri V.K., Wales T.E., Engen J.R., Makriyannis A. (2013). Membrane phospholipid bilayer as a determinant of monoacylglycerol lipase kinetic profile and conformational repertoire. Prot. Sci..

[34] Zhou Q., Lv H., Mazloom A.R., Xu H., Ma’ayan A., Gallo J.M. (2012). Activation of alternate prosurvival pathways accounts for acquired sunitinib resistance in U87MG glioma xenografts. J. Pharmacol. Exp. Ther..

[35] Saleeb R.M., Farag M., Lichner Z., Brimo F., Bartlett J., Bjarnason G., Finelli A., Rontondo F., Downes M.R., Yousef G.M. (2018). Modulating ATP binding cassette transporters in papillary renal cell carcinoma type 2 enhances its response to targeted molecular therapy. Mol. Oncol..

[36] Elmeliegy M.A., Carcaboso A.M., Tagen M., Bai F., Stewart C.F. (2011). Role of ATP-binding cassette and solute carrier transporters in erlotinib CNS penetration and intracellular accumulation. Clin. Cancer Res..

[37] Hu S., Chen Z., Franke R., Orwick S., Zhao M., Rudek M.A., Sparreboom A., Baker S.D. (2009). Interaction of the multikinase inhibitors sorafenib and sunitinib with solute carriers and ATP-binding cassette transporters. Clin. Cancer Res..

[38] Sugawara S., Takayanagi M., Honda S., Tatsuta T., Fujii Y., Ozeki Y., Ito J., Sato M., Hosono A.M. (2020). Catfish egg lectin affects influx and efflux rates of sunitinib in human cervical carcinoma HeLa cells. Glycobiology.

[39] Zimmerman E.I., Hu S., Roberts J.L., Gibson A.A., Orwick S.J., Li L., Sparreboom A., Baker S.D. (2013). Contribution of OATP1B1 and OATP1B3 to the disposition of sorafenib and sorafenib-glucuronide. Clin. Cancer Res..

[40] Jain A., Kameswaran M., Pandey U., Prabhash K., Sarma H.D., Dash A. (2017). (68)Ga labeled Erlotinib: A novel PET probe for imaging EGFR over-expressing tumors. Bioorg. Med. Chem. Lett..

[41] Bauer M., Matsuda A., Wulkersdorfer B., Philippe C., Traxl A., Özvegy-Laczka C., Stanek J., Nics L., Klebermass E.M., Poschner S. (2018). Influence of OATPs on hepatic disposition of erlotinib measured with positron emission tomography. Clin. Pharmacol. Ther..

[42] Altintas I., Heukers R., van der Meel R., Lacombe M., Amidi M., van Bergen En Henegouwen P.M., Hennink W.E., Schiffelers R.M., Kok R.J. (2013). Nanobody-albumin nanoparticles (NANAPs) for the delivery of a multikinase inhibitor 17864 to EGFR overexpressing tumor cells. J. Control. Release.

[43] Dora C.P., Kushwah V., Katiyar S.S., Kumar P., Pillay V., Suresh S., Jain S. (2017). Improved oral bioavailability and therapeutic efficacy of erlotinib through molecular complexation with phospholipid. Int. J. Pharm..

[44] Shi J.F., Sun M.G., Li X.Y., Zhao Y., Ju R.J., Mu L.M., Yan Y., Li X.T., Zeng F., Lu W.L. (2015). A combination of targeted sunitinib liposomes and targeted vinorelbine liposomes for treating invasive breast cancer. J. Biomed. Nanotechnol..

[45] Li F., Mei H., Gao Y., Xie X., Nie H., Li T., Zhang H., Jia L. (2017). Co-delivery of oxygen and erlotinib by aptamer-modified liposomal complexes to reverse hypoxia-induced drug resistance in lung cancer. Biomaterials.

[46] Lakkadwala S., Dos Santos Rodrigues B., Sun C., Singh J. (2020). Biodistribution of TAT or QLPVM coupled to receptor targeted liposomes for delivery of anticancer therapeutics to brain In Vitro and In Vivo. Nanomed. Nanotechnol. Biol. Med..

[47] Saber M.M., Bahrainian S., Dinarvand R., Atyabi F. (2017). Targeted drug delivery of Sunitinib Malate to tumor blood vessels by cRGD-chiotosan-gold nanoparticles. Int. J. Pharm..

[48] Lakkadwala S., Singh J. (2019). Co-delivery of doxorubicin and erlotinib through liposomal nanoparticles for glioblastoma tumor regression using an In Vitro brain tumor model. Colloids Surf. B.

[49] Dora C.P., Trotta F., Kushwah V., Devasari N., Singh C., Suresh S., Jain S. (2016). Potential of erlotinib cyclodextrin nanosponge complex to enhance solubility, dissolution rate, in vitro cytotoxicity and oral bioavailability. Carbohydr. Polym..

[50] Fellmann P., Zachowski A., Devaux P.F., Graham J.M., Higgins J.A. (1994). Synthesis and use of spin-labeled lipids for studies of the transmembrane movement of phospholipids. Biomembrane Protocols: II. Architecture and Function.

[51] Mayer L.D., Hope M.J., Cullis R.P., Janoff A.S. (1985). Solute distributions and trapping efficiencies observed in freeze-thawed multilamellar vesicles. Biochim. Biophys. Acta.

[52] Davis J.H., Jeffrey K.R., Bloom M., Valic M.I., Higgs T.P. (1976). Quadrupolar echo deuteron magnetic resonance spectroscopy in ordered hydrocarbon chains. Chem. Phys. Lett..

[53] Sternin E., Bloom M., Mackay A.L. (1983). De-pake-ing of NMR spectra. J. Magn. Reson..

[54] Lafleur M., Fine B., Sternin E., Cullis P.R., Bloom M. (1989). Smoothed orientational order profile of lipid bilayers by 2H-nuclear magnetic resonance. Biophys. J..

[55] McIntyre J.C., Sleight R.G. (1991). Fluorescence assay for phospholipid membrane asymmetry. Biochemistry.

[56] Pomorski T., Herrmann A., Zachowski A., Devaux P.F., Müller P. (1994). Rapid determination of the transbilayer distribution of NBD-phospholipids in erythrocyte membranes with dithionite. Mol. Membr. Biol..

[57] Greube A., Müller K., Töpfer-Petersen E., Herrmann A., Müller P. (2001). Influence of the bovine seminal plasma protein PDC-109 on the physical state of membranes. Biochemistry.

[58] Langner M., Hui S.W. (1993). Dithionite penetration through phospholipid bilayers as a measure of defects in lipid molecular packing. Chem. Phys. Lipids.

[59] Tannert A., Töpfer-Petersen E., Herrmann A., Müller K., Müller P. (2007). The lipid composition modulates the influence of the bovine seminal plasma protein PDC-109 on membrane stability. Biochemistry.

