# Supplementary material for: Impact of Selected Small-Molecule Kinase Inhibitors on Lipid Membranes
Source: Pharmaceuticals (Basel). 2021 Jul 29;14(8):746. doi: 10.3390/ph14080746 (PMC8401620; doi:10.3390/ph14080746)
Supplement: Supplementary file 1 [file pharmaceuticals-14-00746-s001.zip › pharmaceuticals-1304568-supplementary.pdf]

Supplementary Materials

Impact of Selected Small-Molecule Kinase Inhibitors on Lipid Membranes

Meike Luck, Markus Fischer, Maximilian Werle, Holger A. Scheidt, Peter Müller

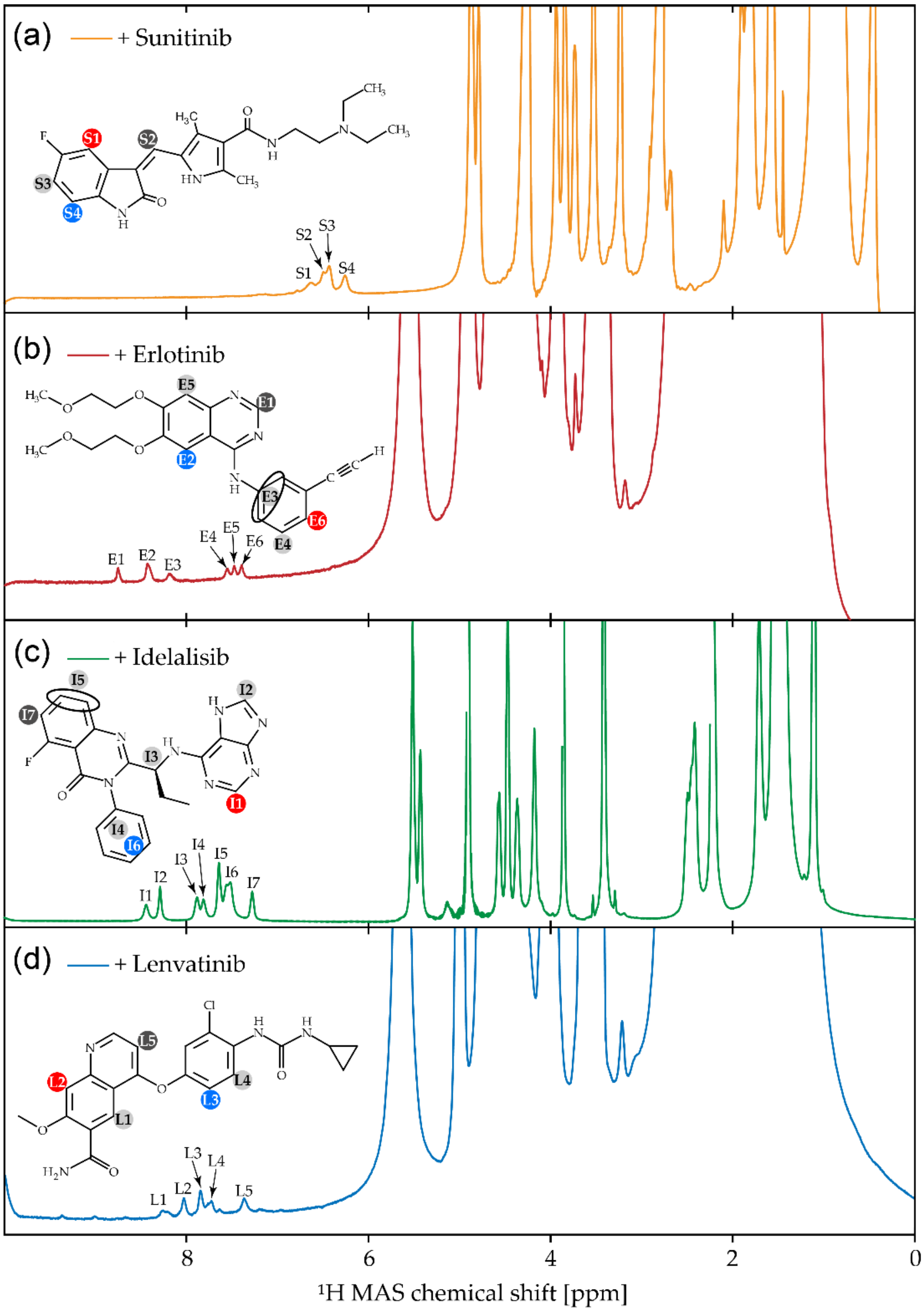

Figure S1. ^{1}H MAS NMR spectra of POPC in the presence of 20 mol% of each inhibitor as well as signal assignment of the relevant inhibitor protons to the spectral peaks. All spectra were acquired at a MAS frequency

of 6000 Hz and a temperature of 303 K. The peak assignment was done with the help of Spinus NMR (http://neural.dq.fct.unl.pt/spinus/).

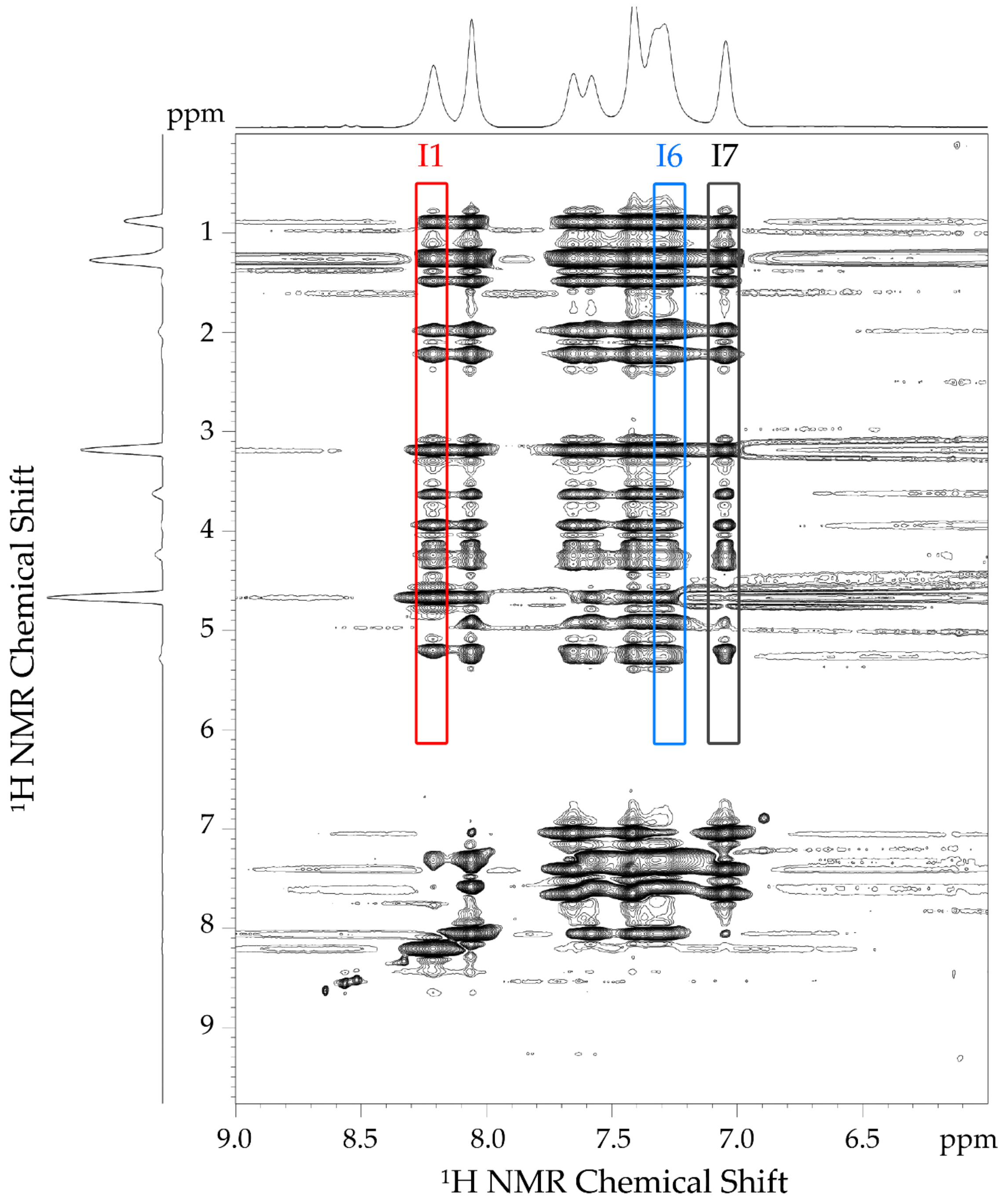

Figure S2. Counter plot of the aromatic region of the ^{1}H MAS NOESY spectrum of 20 mol% idelalisib in a POPC membrane at a mixing time of 300 ms. The cross peaks between protons of idelalisib and POPC used for quantitative analysis are highlighted. All cross peaks have positive intensity.

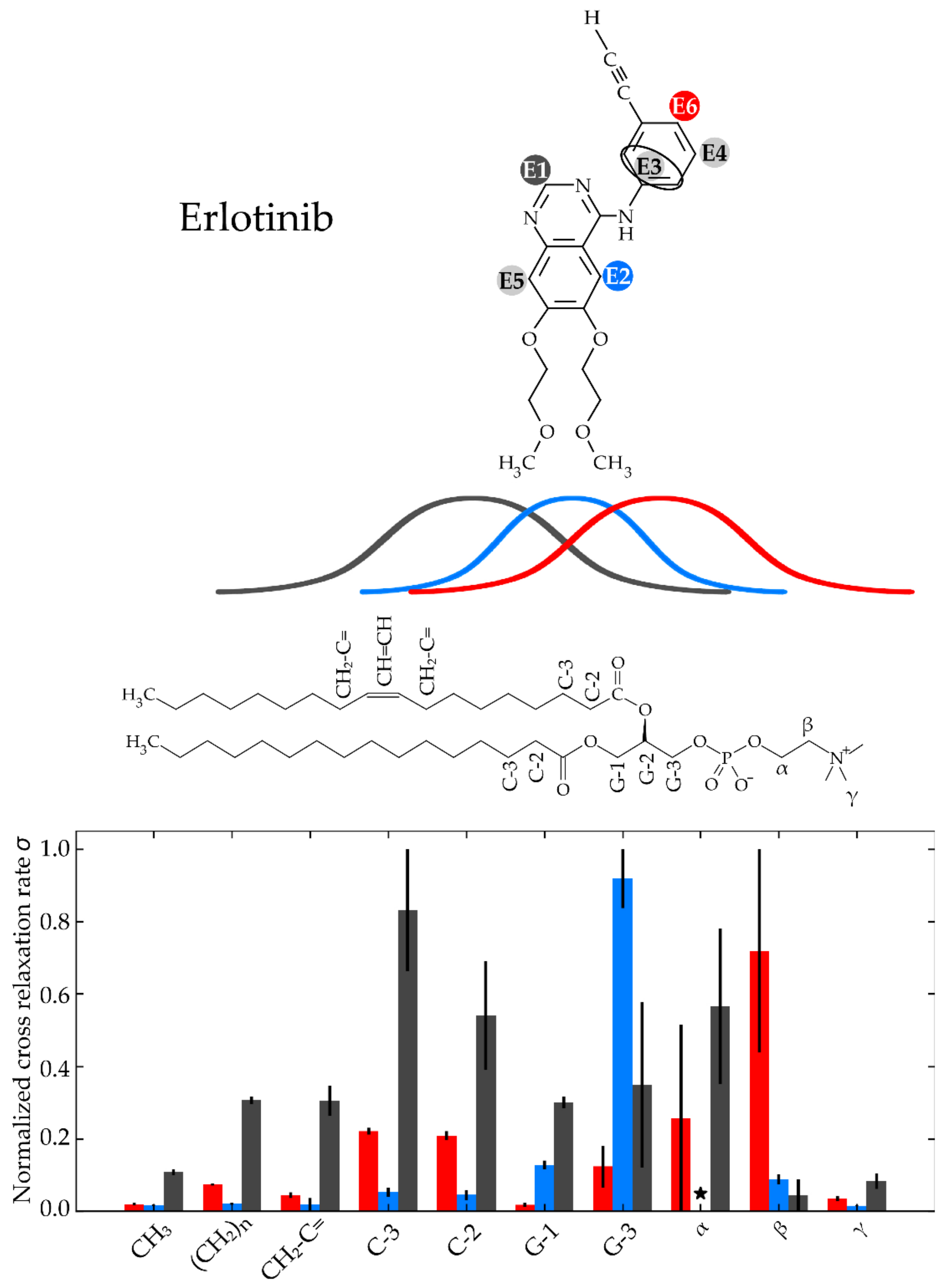

Figure S3. Normalized cross relaxation rates of the protons (E1, E2, E6) of erlotinib with the proton groups of the POPC membrane. Erlotinib adopts an average localisation in the glycerol region. The benzene like ring points towards the aqueous phase, while the short chains will most likely orient themselves towards the membrane center. A star marks cross correlation rates that were ignored due to low signal-to-noise ratio.

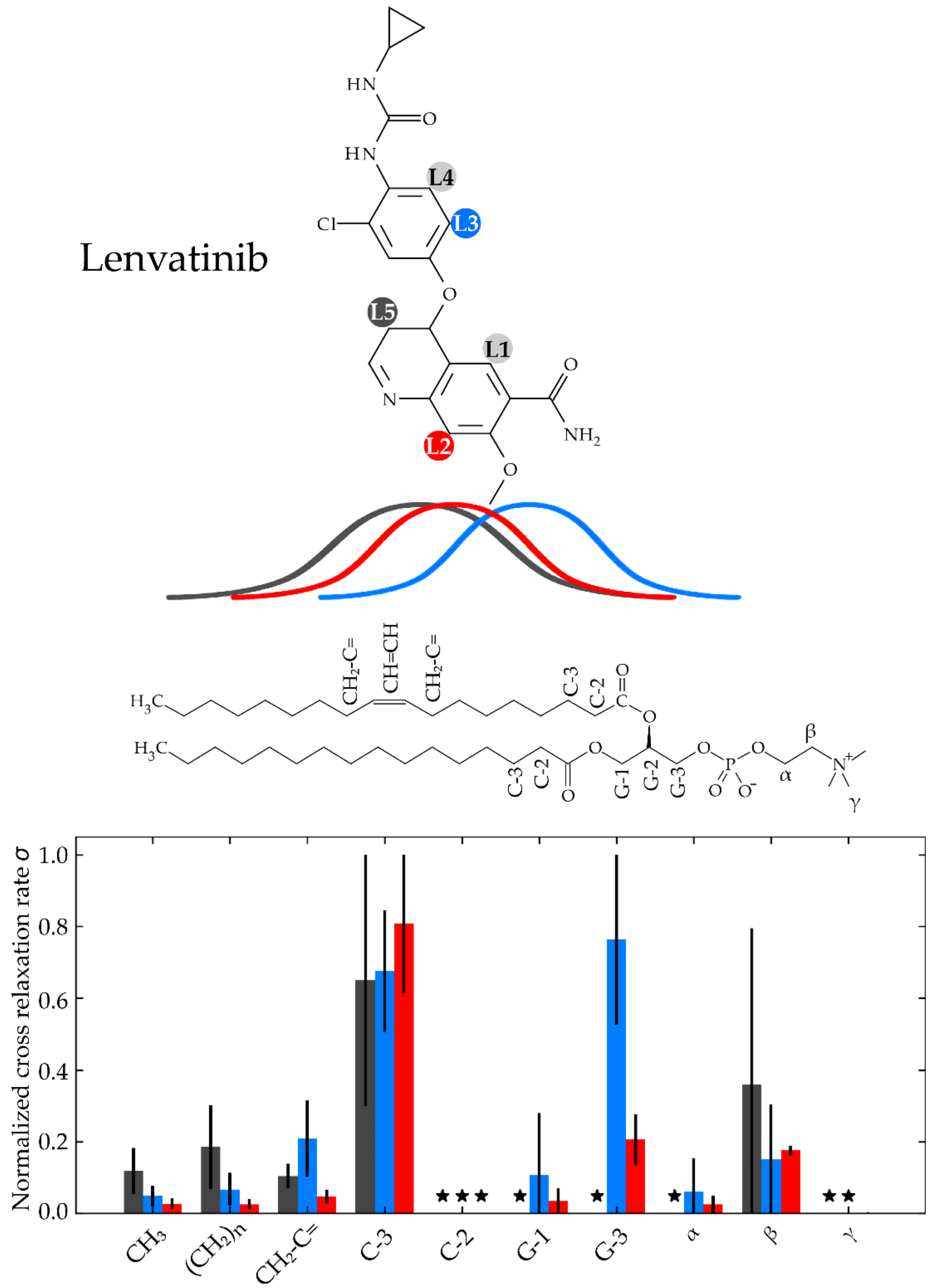

Figure S4. Normalized cross relaxation rates of the protons (L2, L3, L5) of lenvatinib with the proton groups of the POPC membrane. The protons L1 and L4 don't produce cross peaks, and the stars indicate cross relaxation rates that could not be computed, due to low signal-to-noise ratio. Lenvatinib is localised in the glycerol region of the membrane with no inherent preference towards the aqueous phase or core.

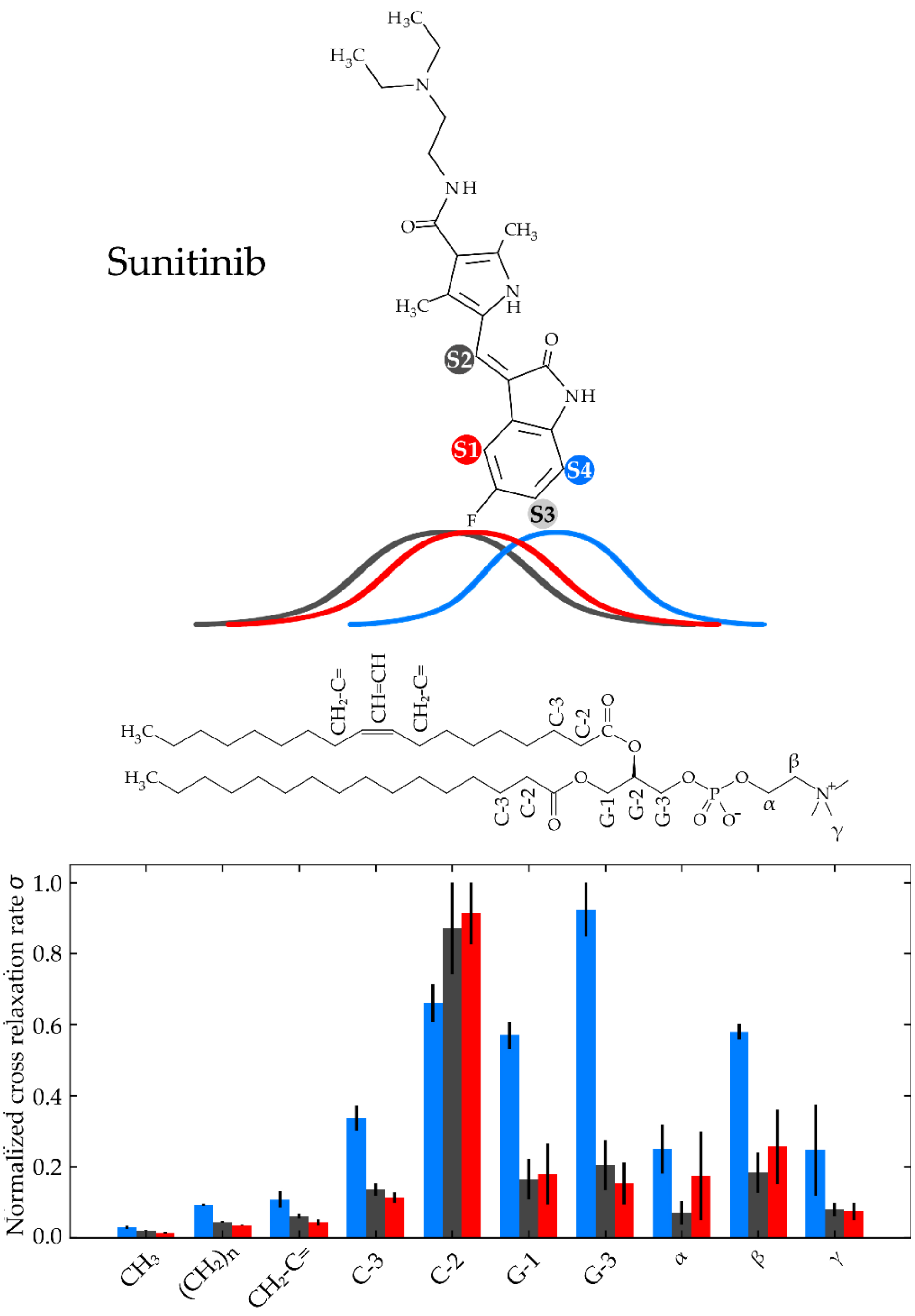

Figure S5. Normalized cross relaxation rates of the protons (S1, S2, S4) of sunitinib with the proton groups of the POPC membrane. The indane-like portion of the molecule is localised in the glycerol region of the membrane, with a slight preference towards the aqueous phase. It is reasonable to assume, that the methyl prong points inwards.

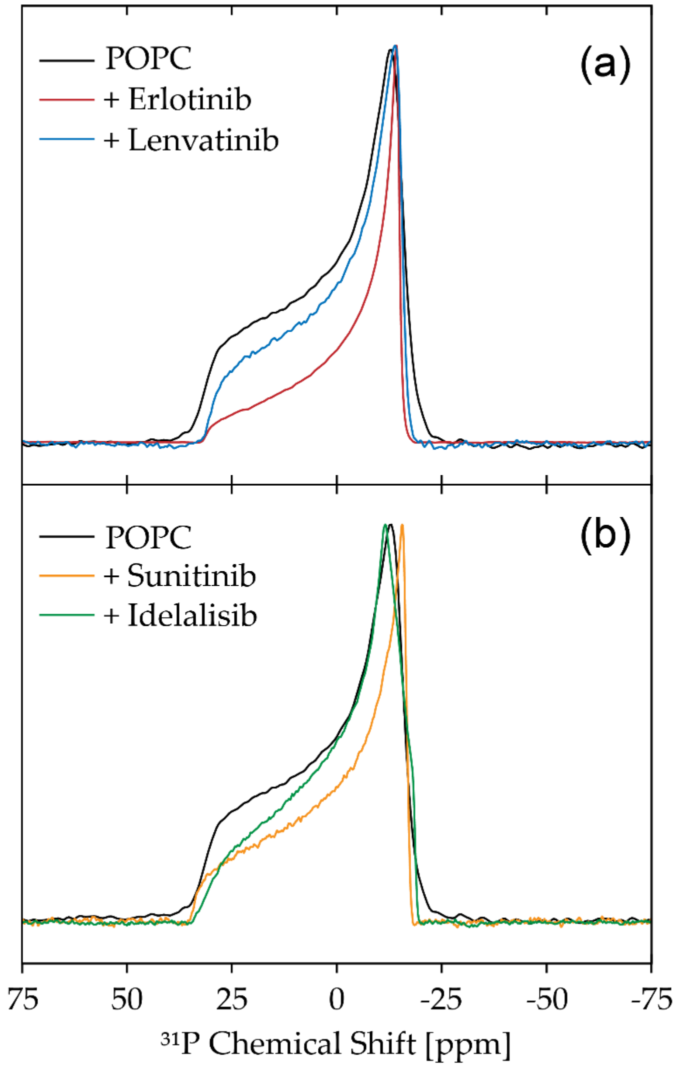

Figure S6. ^{31}P NMR powder spectra of both pure POPC and within and without presence of 20 mol% of each inhibitor. The line shapes indicate that the POPC vesicles retain their lamellar phase even in the presence of the inhibitors, and the CSA values are only slightly varied. Erlotinib has a rather pronounced orientation effect.

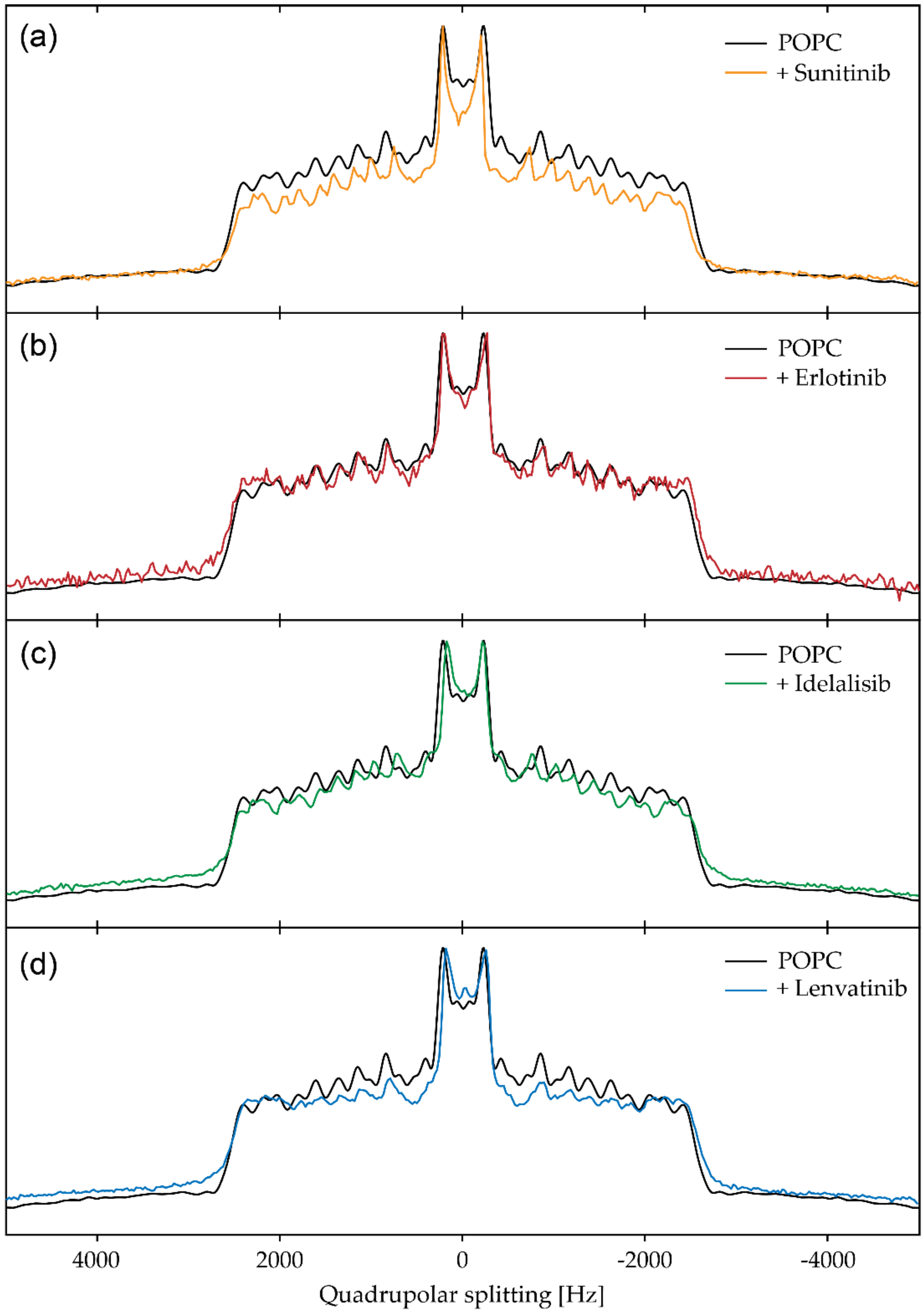

Figure S7. Static ^{2}H NMR powder spectra of both pure POPC-d_{31}, and POPC-d_{31} in presence of 20 mol% inhibitor. All spectra were recorded at 303 K.

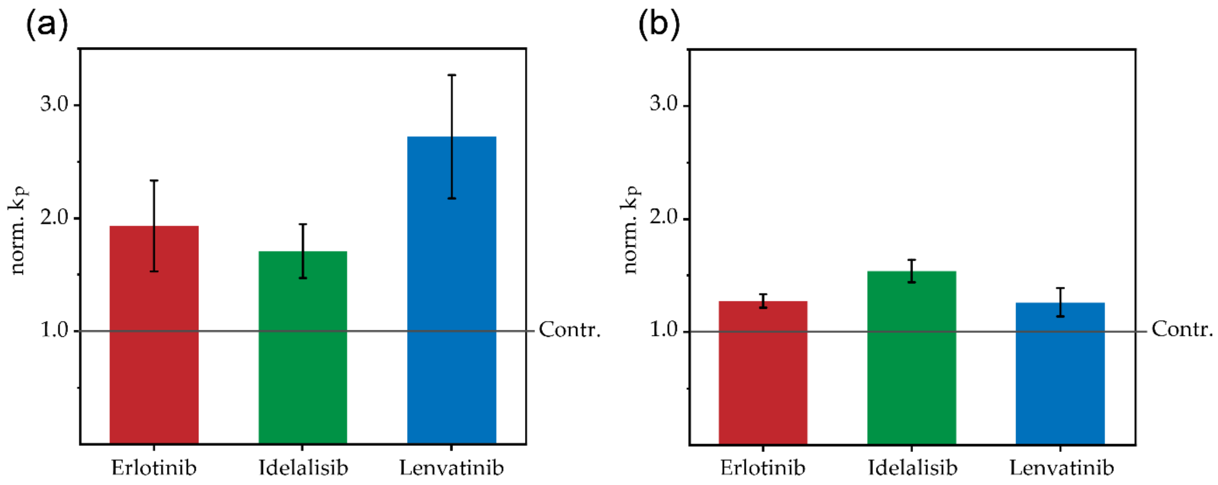

Figure S8. Influence of small-molecule kinase inhibitors on membrane integrity. The membrane permeation of the anion dithionite was measured in the absence and presence of drugs by fluorescence spectroscopy. From experimental reduction kinetics of NBD-PC upon addition of dithionite measured at 37°C, the rate constants (k_P) for dithionite permeation was determined. The k_P values in in the presence of respective drugs (L:D = 2.5:1) were normalized to those determined in the absence of drugs (only addition of DMSO). The mean +/- SE of 3 independent experiments are shown for POPC LUVs (a) and for POPC/cholesterol (4:1) LUVs (b).
